# Supplementary material for: Engineering SARS-CoV-2 specific cocktail antibodies into a bispecific format improves neutralizing potency and breadth
Source: Nat Commun. 2022 Sep 22;13:5552. doi: 10.1038/s41467-022-33284-y (PMC9499943; doi:10.1038/s41467-022-33284-y)
Supplement: Supplementary file 1 — Supplementary Information [file 41467_2022_33284_MOESM1_ESM.pdf]

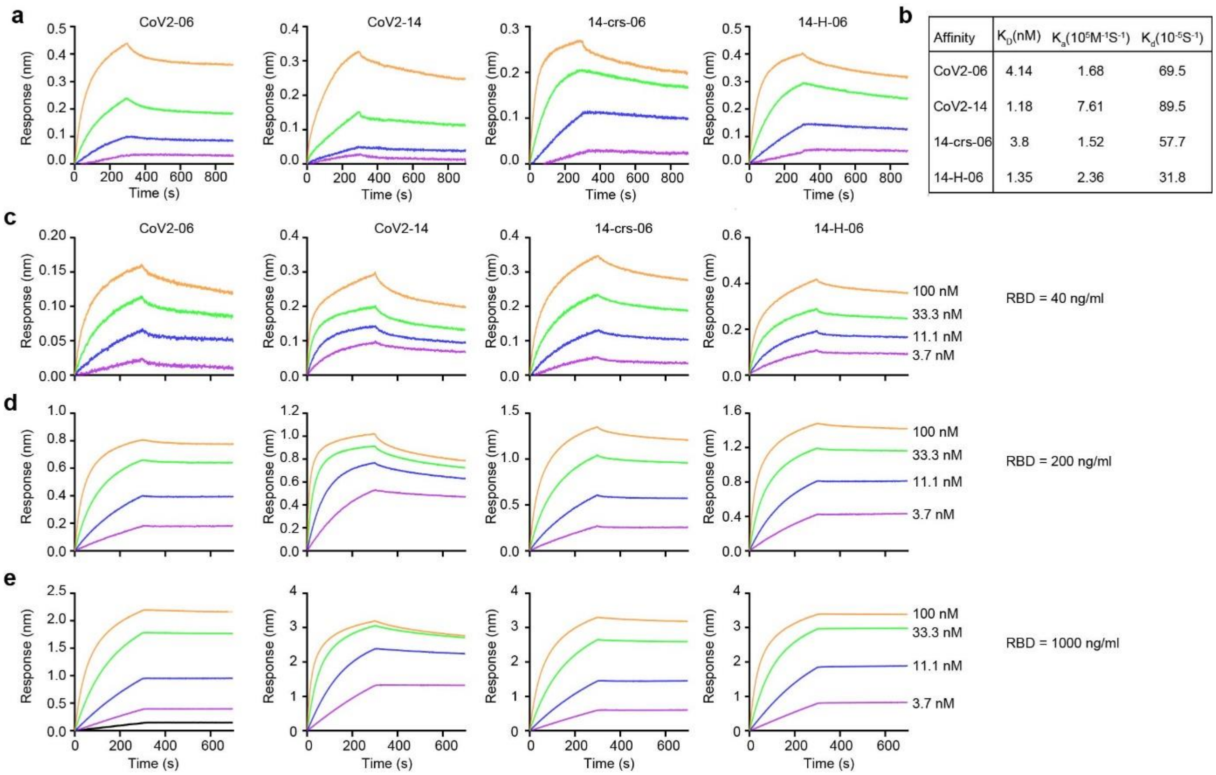

Affinity	K_D(nM)	K_a($10^5 M^{-1} S^{-1}$)	K_d($10^{-5} S^{-1}$)
CoV2-06	4.14	1.68	69.5
CoV2-14	1.18	7.61	89.5
14-crs-06	3.8	1.52	57.7
14-H-06	1.35	2.36	31.8

Supplementary Fig. 1. Kinetic bindings of the BLI-based affinity and avidity assays. **a**, The sensorgrams of affinity binding for indicated antibodies. Antibodies were immobilized onto the protein A biosensors and the RBD-His was in solutions. **b**, Summary of the affinity binding (K_D), the association (K_{on}) and the dissociation (K_{dis}) parameters. **c-e**, The sensorgrams in the avidity binding for indicated antibodies. The RBD-His was immobilized onto the Ni-NTA biosensors at concentrations of 40 ng/ml (**c**), 200 ng/ml (**d**) and 1000 ng/ml (**e**) and indicated antibodies were in solutions.

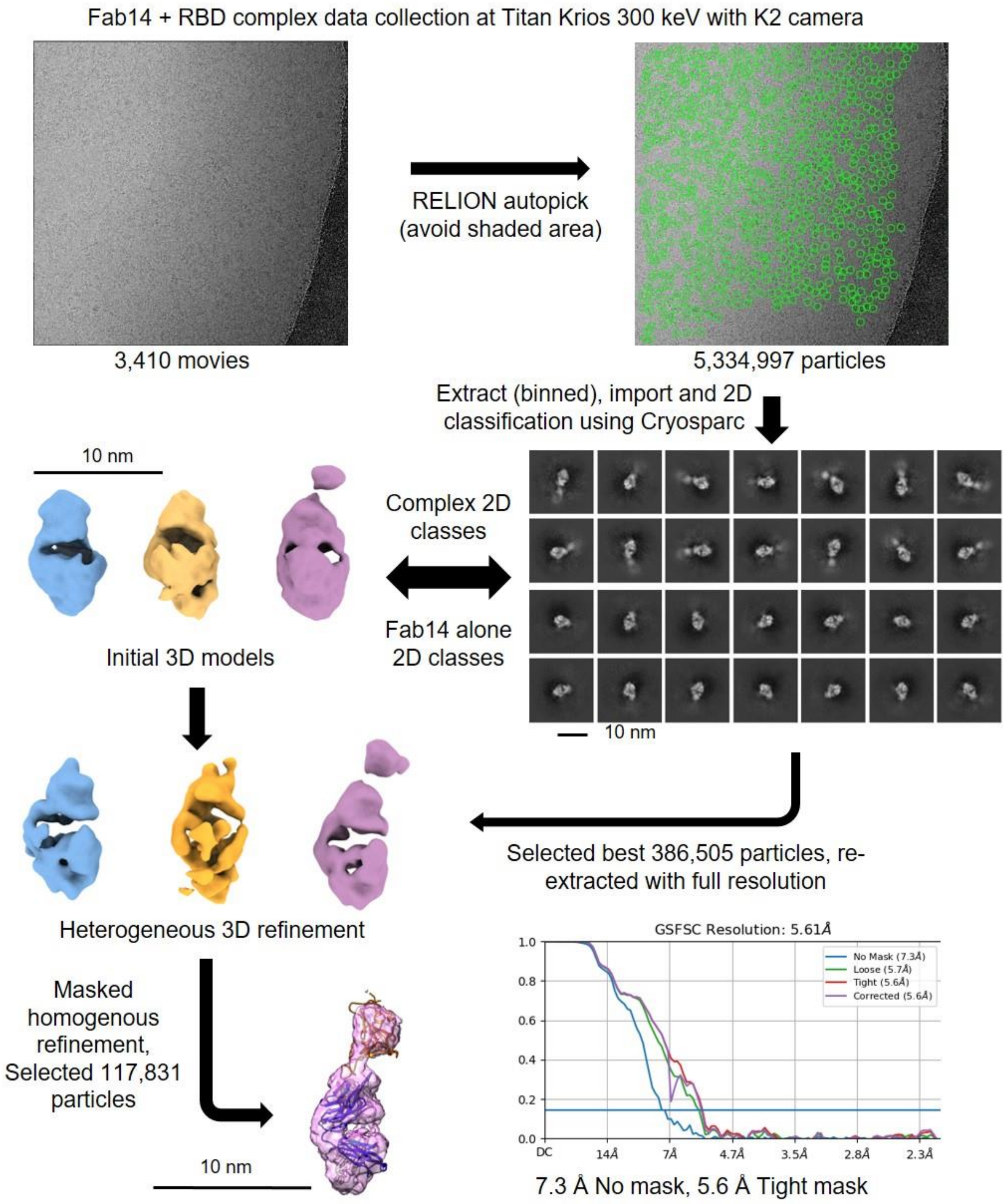

Supplementary Fig. 2. Fab14+RBD complex cryo-EM data processing workflow.

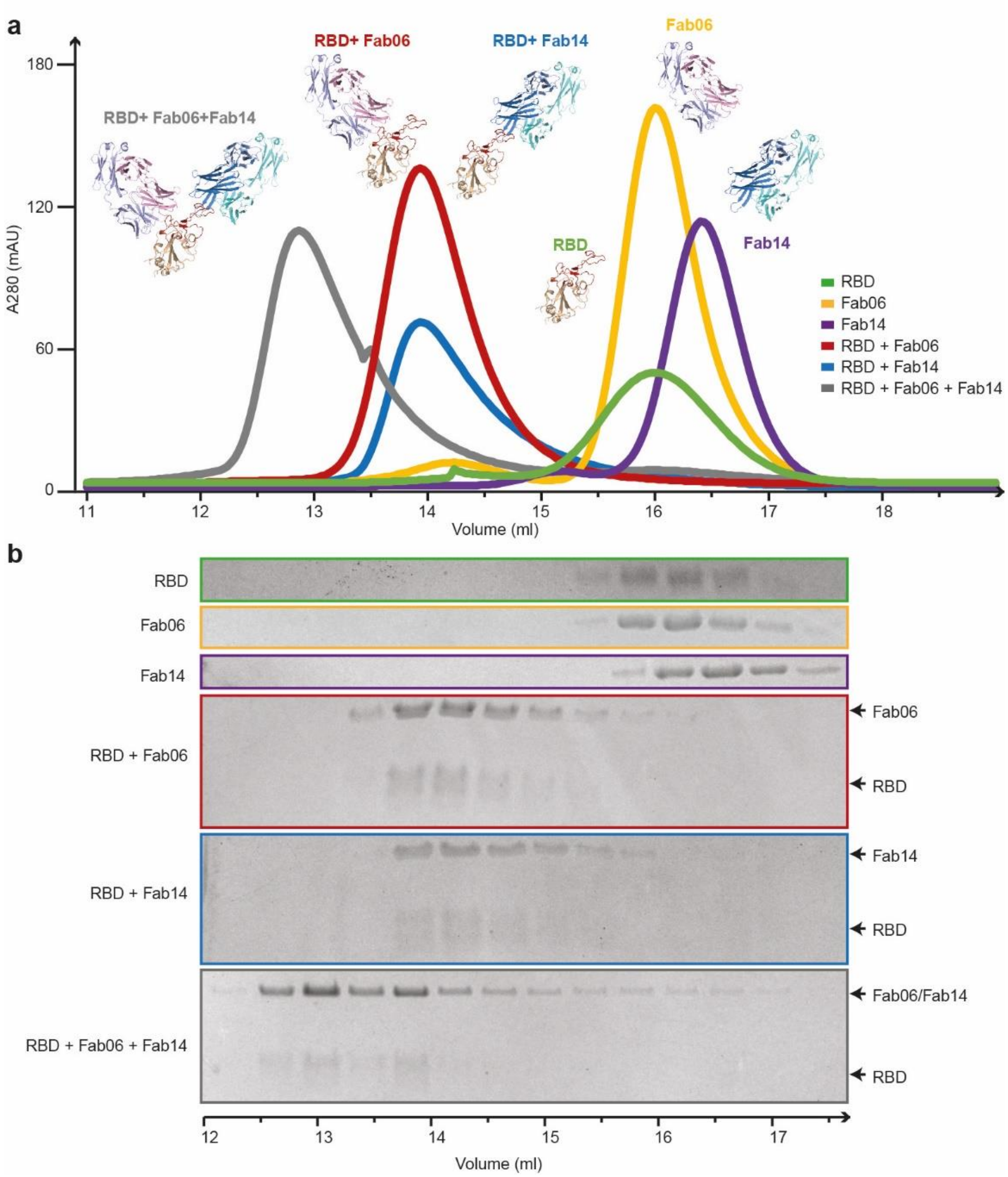

Supplementary Fig. 3. Complex formation between RBD, Fab06, and Fab14. (**a**) The size-exclusion chromatography (SEC) analysis of indicated Fab, RBD and Fab/RBD complexes. (**b**) The non-reducing SDS-PAGE analysis of the fractions collected from the SEC analysis. The image is from one experiment.

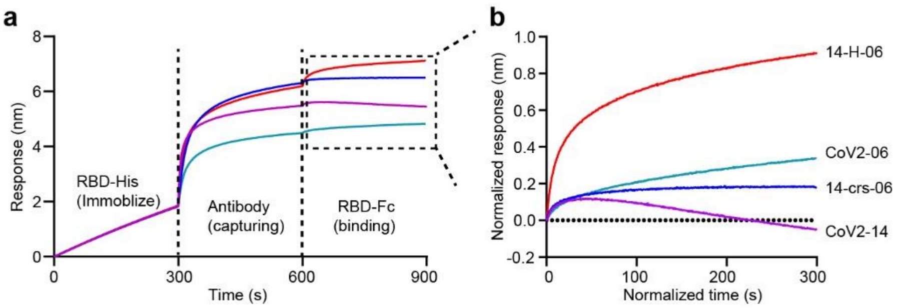

Supplementary Fig. 4. Multivalent binding to RBD by 14-H-06. Simultaneous binding of antibodies to multiple RBDs was determined by a BLI sandwich assay. **a**, The sensorgrams showing the immobilization of RBD-His for 300s, the capturing of indicated antibodies for 300s and the following binding by RBD-Fc for 300s. The binding to RBD-Fc shown in the dashed box was normalized and shown in the panel **b**.

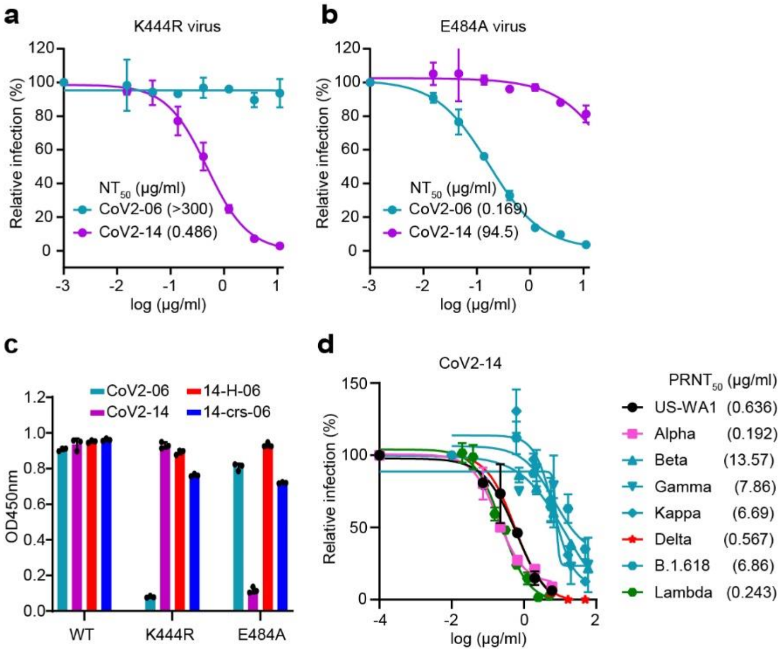

Supplementary Fig. 5. Additional binding and neutralizing characterizations of antibody against the variants. (**a-b**) Neutralizations of SARS-CoV-2 virus with K444R mutation (**a**) and E484A mutation (**b**) by CoV2-06 and CoV2-14. The assay is based on the mNeonGreen reporter virus and the NT_{50} values are labeled. **c**, ELISA binding to the WT RBD and the K444R and E484A RBD mutants by indicated antibodies. **d**, PRNT of CoV2-14 against the SARS-CoV-2 US-WA1 strain and indicated SARS-CoV-2 variants. The $PRNT_{50}$ values are labeled. Data are from two replicates for a, b and d and from three replicates for c.

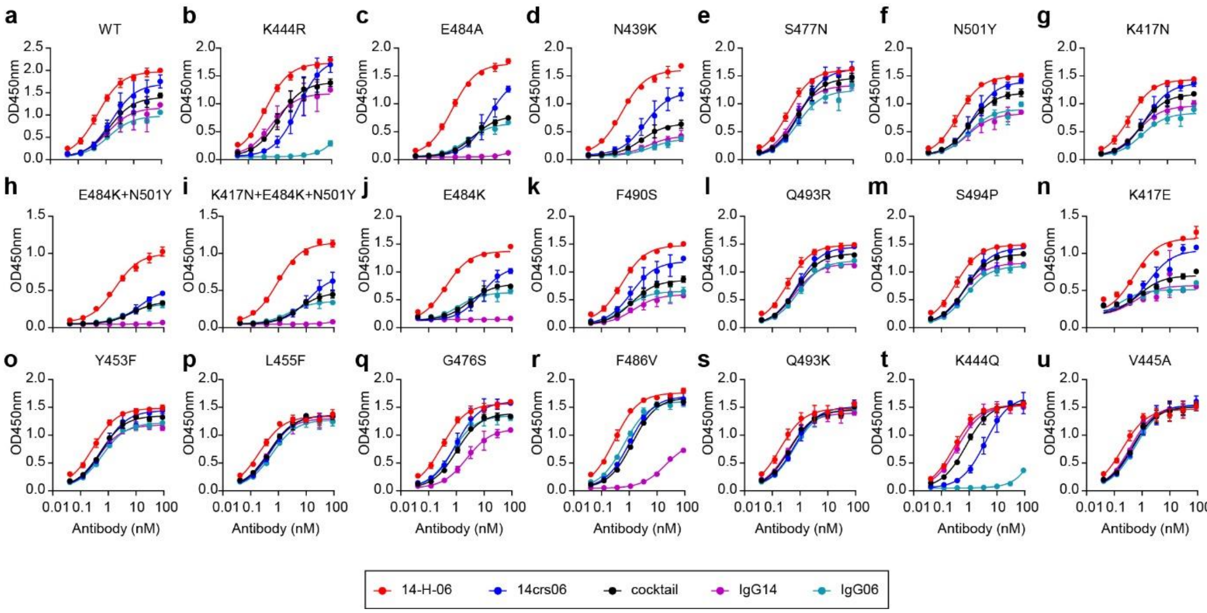

Supplementary Fig. 6. ELISA bindings of bsAbs, individual antibodies and the cocktail to wild type and mutant RBDs. **a-u**, ELISA titrations of indicated antibodies to immobilized WT RBD and RBD mutants. Data points are from duplicate wells.

a

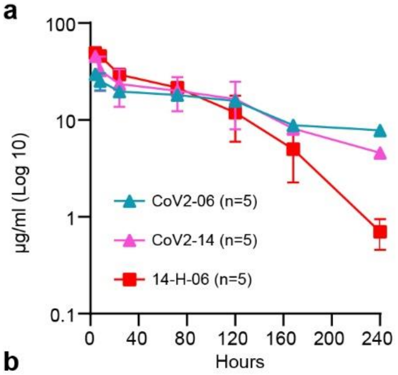

b

	CoV2-06	CoV2-14	14-H-06
T1/2 (hr.)	137.40	74.72	29.02
AUC (hr/ug/ml)	3438.63	3667.56	3533.27

Supplementary Fig. 7. Antibody pharmacokinetics in mice. a, The serum concentrations of injected antibodies at multiple time points (4, 8, 24, and 72 hours 5, 7, 10 days) post injection were quantified by ELISA. **b**, Pharmacokinetics parameters were calculated by non-compartmental analysis using Phoe^{ni}x 64 WinNonlin (8.3.3.33) software (Certara).

Table S1. Data collection and refinement statistics for crystal structures

	**Fab06-RBD Complex**	**Fab14**
Wavelength (Å)	0.9464	0.9464
Resolution range (Å)[a]	47.55 - 2.89 (2.993 - 2.89)	48.03 - 2.46 (2.548 - 2.46)
Space group	P $2_1 2_1 2$	C2
Unit cell a,b,c (Å)	50.17, 266.35, 112.61	77.7, 71.22, 96.76
α,β,γ (°)	90, 90, 90	90, 99.07, 90
Total reflections	363812 (35178)	130818 (11319)
Unique reflections	34882 (3389)	18867 (1696)
Multiplicity	10.4 (10.4)	6.9 (6.7)
Completeness (%)	99.81 (98.86)	98.83 (89.35)
Mean I/sigma(I)	8.31 (1.08)	13.60 (1.81)
Wilson B-factor	63.19	53.73
R_{merge}	0.3153 (2.504)	0.1281 (1.076)
R_{meas}	0.3316 (2.632)	0.1385 (1.165)
R_{pim}	0.1006 (0.7924)	0.05213 (0.4418)
$CC_{1/2}$	0.994 (0.48)	0.997 (0.765)
CC*	0.998 (0.806)	0.999 (0.931)
Reflections used in refinement	34874 (3388)	18855 (1694)
Reflections used for R-free	1743 (170)	943 (85)
R_{work}[b]	0.2287 (0.3623)	0.2104 (0.3511)
R_{free}[b]	0.2694 (0.4140)	0.2563 (0.3683)
$CC_{(work)}$	0.938 (0.656)	0.948 (0.795)
$CC_{(free)}$	0.888 (0.374)	0.907 (0.788)
Number of non-hydrogen atoms	9536	3384
protein	9411	3310
NAG	28	
water	97	74
Protein residues	1242	438
RMS(bonds) (Å)	0.012	0.010
RMS(angles) (°)	1.55	1.27
Ramachandran favored (%)	95.43	95.39
Ramachandran allowed (%)	4.57	4.61
Ramachandran outliers (%)	0.00	0.00
Rotamer outliers (%)	0.66	0.00
Clashscore	13.06	8.74
Average B-factor	67.21	57.01
protein	67.34	57.00
water	49.33	57.17

Table S2. Engineered mutations in the spike region of recombinant SARS-CoV-2 variants.

**Variants**	**Spike mutations**
Alpha (B.1.1.7)	Δ69-70, Δ145, N501Y, A570D, D614G, P681H, T716I, S982A, and D1118H
Beta (B.1.351)	D80A, D215G, Δ242-244, K417N, E484K, N501Y, D614G, and A701V
Gamma (P.1)	L18F, T20N, P26S, D138Y, R190S, K417T, E484K, N501Y,D614G, H655Y, T1027I, and V1176F
Kappa (B.1.617.1)	G142D, E154K, L452R, E484Q, D614G, P681R, Q1071H, and H1101D
Delta (B.1.617.2)	T19R, G142D, L452R, T478K, D614G, P681R, and D950N
Lambda	G75V, T76I, Δ246-252, D253N, L452Q, F490S, D614G, and T859N
B.1.618	H49Y, Δ145-146, E484K, and D614G
Omicron (B.1.1.529)	A67V, del69-70, T95I, Δ142-144, Y145D, del211, L212I, ins214EPE, G339D, S371L, S373P, S375F, K417N, N440K, G446S, S477N, T478K, E484A, Q493R, G496S, Q498R, N501Y, Y505H, T547K, D614G, H655Y, N679K, P681H, N764K, D796Y, N856K, Q954H, N969K, L981F
