# Supplementary material for: Engineering SARS-CoV-2 specific cocktail antibodies into a bispecific format improves neutralizing potency and breadth
Source: Nat Commun. 2022 Sep 22;13:5552. doi: 10.1038/s41467-022-33284-y (PMC9499943; doi:10.1038/s41467-022-33284-y)
Supplement: Supplementary file 2 — Reporting Summary [file 41467_2022_33284_MOESM2_ESM.pdf]

nature portfolio

Corresponding author(s): Zhiqiang An

Last updated by author(s): Sep 1, 2022

Reporting Summary

Nature Portfolio wishes to improve the reproducibility of the work that we publish. This form provides structure for consistency and transparency in reporting. For further information on Nature Portfolio policies, see our Editorial Policies and the Editorial Policy Checklist.

Statistics

For all statistical analyses, confirm that the following items are present in the figure legend, table legend, main text, or Methods section.

n/a	Confirmed	
☐	☒	The exact sample size (n) for each experimental group/condition, given as a discrete number and unit of measurement
☐	☒	A statement on whether measurements were taken from distinct samples or whether the same sample was measured repeatedly
☐	☒	The statistical test(s) used AND whether they are one- or two-sided<br>*Only common tests should be described solely by name; describe more complex techniques in the Methods section.*
☒	☐	A description of all covariates tested
☐	☒	A description of any assumptions or corrections, such as tests of normality and adjustment for multiple comparisons
☐	☒	A full description of the statistical parameters including central tendency (e.g. means) or other basic estimates (e.g. regression coefficient) AND variation (e.g. standard deviation) or associated estimates of uncertainty (e.g. confidence intervals)
☐	☒	For null hypothesis testing, the test statistic (e.g. F, t, r) with confidence intervals, effect sizes, degrees of freedom and P value noted<br>*Give P values as exact values whenever suitable.*
☒	☐	For Bayesian analysis, information on the choice of priors and Markov chain Monte Carlo settings
☒	☐	For hierarchical and complex designs, identification of the appropriate level for tests and full reporting of outcomes
☒	☐	Estimates of effect sizes (e.g. Cohen's d, Pearson's r), indicating how they were calculated

Our web collection on statistics for biologists contains articles on many of the points above.

Software and code

Policy information about availability of computer code

Data collection	Octet Data Acquisition 9.0 was used to collect affinity and avidity data, antibody competition data<br>SoftMax Pro 6.5.1 was used to collect ELISA data<br>UNICORN V7.0 was used to collect the SEC data<br>Gen5™ Microplate Reader and Imager was used to collect virus infection and neutralization data<br>X-ray diffraction data was collected by Macromolecular Crystallography II (MXII)<br>The image of virus infection was collected by a Cytation 7 multimode reader<br>Molecular modeling was performed by the HADDOCK 2.4 webserver and MODELLER<br>MD simulations was performed using NAMD 2.12<br>The Cryo-EM particles were analyzed using cyroSPARC v3.2 and the images were corrected with RELION's implementation of the MotionCor2 algorithm
Data analysis	Structure refinement was performed using both Buster and Phenix Refine interspersed with manual model correction using Coot.<br>Structure analysis and image production were made using PyMOL<br>The GraphPad prism 8 Software was used for dataset plotting and statistic analysis<br>The Phoenix 64 WinNonlin (8.3.3.33) software (Certara) was used for half-life analysis

For manuscripts utilizing custom algorithms or software that are central to the research but not yet described in published literature, software must be made available to editors and reviewers. We strongly encourage code deposition in a community repository (e.g. GitHub). See the Nature Portfolio guidelines for submitting code & software for further information.

Data

Policy information about availability of data

All manuscripts must include a data availability statement. This statement should provide the following information, where applicable:

- Accession codes, unique identifiers, or web links for publicly available datasets
- A description of any restrictions on data availability
- For clinical datasets or third party data, please ensure that the statement adheres to our policy

Data associated with figures are available from the corresponding authors upon reasonable request. Structures and structure factors reported in this work have been deposited with the PDB with accession codes 7WPH (Fab-06-RBD complex) and 7WPV (Fab-14).
Data associated with figures are available are provided and publicly available.
Data availability: Data associated with figures are available from the corresponding authors upon reasonable request. Structures and structure factors reported in this work have been deposited with the PDB with accession codes 7WPH (Fab06/RBD complex) and 7WPV (Fab14), and 7XXL (Fab14/RBD complex) with the density map (EMD-33506). Source data are provided with this paper.

Field-specific reporting

Please select the one below that is the best fit for your research. If you are not sure, read the appropriate sections before making your selection.

[x] Life sciences [ ] Behavioural & social sciences [ ] Ecological, evolutionary & environmental sciences

For a reference copy of the document with all sections, see nature.com/documents/nr-reporting-summary-flat.pdf

Life sciences study design

All studies must disclose on these points even when the disclosure is negative.

Sample size	The group sizes for in vitro assays (e.g. ELISA, BLI, neutralization and flow cytometry) were selected based on prior knowledges in our previous publications (PMID: 33106671; 31213474). The group sizes for in vivo assays were selected on the basis of pilot studies and prior knowledges (PMID: 33106671).
Data exclusions	no
Replication	Replicates were used in the experiments as noted in the methods, figure legends and text.
Randomization	Mice were the same background , age- and gender-matched and randomized by animal research personnel at UTMB prior to experiment. Randomizations are irrlevant to in vitro cell line based assays or biochemical assays because they from a same stock.
Blinding	No blinding was performed due to safety considerations regarding infected animals and cell culture.

Reporting for specific materials, systems and methods

We require information from authors about some types of materials, experimental systems and methods used in many studies. Here, indicate whether each material, system or method listed is relevant to your study. If you are not sure if a list item applies to your research, read the appropriate section before selecting a response.

Materials & experimental systems

n/a	Involved in the study	
[ ]	[x]	Antibodies
[ ]	[x]	Eukaryotic cell lines
[x]	[ ]	Palaeontology and archaeology
[ ]	[x]	Animals and other organisms
[x]	[ ]	Human research participants
[x]	[ ]	Clinical data
[x]	[ ]	Dual use research of concern

Methods

n/a	Involved in the study	
[x]	[ ]	ChIP-seq
[x]	[ ]	Flow cytometry
[x]	[ ]	MRI-based neuroimaging

Antibodies

Antibodies used	Goat Anti-Human IgG, F(ab')2 fragment specific, poly-clonal, Jackson Immuno Research, #109-035-006, 1:5000<br>All the SARS-CoV-2 antibodies were generated in house

Validation	Goat Anti-Human IgG, F(ab')2 fragment was tested by the manufacture to demonstrate its ability to detect the F(ab')2 fragment. 1:5000

Eukaryotic cell lines

Policy information about cell lines

Cell line source(s)	Expi293 cells, Thermo Fisher, Cat#A14527; Vero-E6, ATCC, CRL-1586
Authentication	The Expi 293, Vero-E6 and HEK-293T cells were previously reported but not authenticated by us.
Mycoplasma contamination	All cell lines were mycoplasma negative
Commonly misidentified lines (See ICLAC register)	none

Animals and other organisms

Policy information about studies involving animals; ARRIVE guidelines recommended for reporting animal research

Laboratory animals	Ten- to twelve-week old female BALB/c mice were purchased from Charles River Laboratories. Eight-to ten-week-old female K18-hACE2 mice were ordered from The Jackson Laboratory. All animals were maintained in SealsafeTM HEPA-filtered air in/out units
Wild animals	none
Field-collected samples	none
Ethics oversight	Mice were maintained at University of Texas Medical Branch at Galveston in accordance with the guidelines of the Institutional Animal Care and use Committee (IACUC) under the protocol 1802011

Note that full information on the approval of the study protocol must also be provided in the manuscript.
